# Network Coding for Efficient Video Multicast in Device-to-Device Communications

**DOI:** 10.3390/s20082254

**Published:** 2020-04-16

**Authors:** Lei Wang, Yulong Li, Bo Pan, Qiuwei Wu, Jun Yin, Lijie Xu

**Affiliations:** 1 Jiangsu Key Laboratory of Big Data Security and Intelligent Processing, Nanjing University of Posts and Telecommunications, Nanjing 210023, China; lyl123456xx@163.com; 2 Jiaxing Guowangtong New Energy Technology Co., Ltd., Jiaxing 314000, China; panbo1@sgitg.sgcc.com.cn (B.P.); quiwudtu@gmail.com (Q.W.); 3 School of Computer, Nanjing University of Posts and Telecommunications, Nanjing 210023, China; junyin@njupt.edu.cn (J.Y.); ljxu@njupt.edu.cn (L.X.)

**Keywords:** Network Coding, Video Distribution, Reliability, Multicast, Load Balancing

## Abstract

Device-to-Device (D2D) communication is one of the critical technologies for the fifth-generation network, which allows devices to communicate directly with each other while increasing transmission rate, but this communication is vulnerable to interference. When video transmission is carried out in an environment with interference, problems such as high packet loss rate, poor quality of the video, and blurred screen may exist. These problems can be effectively solved by redundant coding operations at the source node, but the extra coding operation imposes a heavy computational burden on the source node. In order to alleviate the computational overhead of the source node, reduce transmission delay, and guarantee transmission quality, this paper proposes an efficient video multicast transmission scheme based on Random Linear Network Coding (RLNC) in D2D networks. In the scheme, the receiving devices in the transmission participate in the process of generating repair packets that are used to remedy the loss of encoded packets during transmission. The source node multicasts the encoded video file. The receiving nodes re-encode the received data packets with RLNC and then send them to the network again. The nearby nodes can decode the original data through the encoded or re-encoded data packets. The performance of the proposed scheme is evaluated through both simulation and real experiments. The experimental results show that compared with the traditional RLNC scheme, this scheme could balance the computation overhead of the mobile devices and reduce the encoding and decoding delay by about 8%. When the packet loss rate is high, the proposed scheme can obtain better video quality than the traditional replication-based scheme.

## 1. Introduction

Device-to-Device (D2D) communication is one of the critical supporting technologies for the fifth-generation mobile communication system [1,2,3]. When the mobile devices are close to each other, they are allowed to conduct direct communication without the support of the base station [4,5], which could significantly increase the bandwidth efficiency. Nowadays, people’s demand for mobile video services is growing rapidly, which brings a great burden to base stations, and the characteristics of D2D communication technology bring hope to reduce the burden of base stations. Therefore, in recent years, there have been more and more studies combining D2D communication technology with video services [6,7,8].

However, the mobile devices in D2D communications may suffer from interference from the base station and other devices working in the same frequency, which will reduce the transmission reliability.

The unreliable transmission will reduce the user experience when transmitting video. During the video transmission process, various problems, such as loss, reversal, and repetition of video frames, may occur. These problems cause abnormal situations at receiving devices, which results in blurriness, crash, green screen, and other problems during video play. The technologies of multicast and broadcast are always used in practical networks because they are faster and more convenient for one-to-many transmission and can significantly improve the throughput of overall networks. However, in such networks, the problems will be more serious [9,10]. The traditional retransmission mechanism can improve transmission reliability, but it will increase transmission overhead and increase the transmission delay. In the communication environment with interference, it is necessary to improve the transmission efficiency [11]. The technology of network coding had provided a new idea to improve this problem [12].

The network coding theory was proposed by Alshwede et al. [13], and they showed that the network throughput could be effectively improved through data re-encoding operation at the intermediate nodes of the networks. Network coding has changed the opinion that the intermediate nodes in the network can only forward data, and it is unfeasible to conduct any additional operation. Network coding has attracted attention from many researchers [14,15]. Li et al. [16] proposed linear network coding and proved that linear network coding could achieve maximum multicast capacity. Köetter et al. [17] extended network coding to an arbitrary network. In order to solve the coding problem in a distributed network environment, Ho et al. [18] proposed Random Linear Network Coding (RLNC), which is considered to be feasible in practical applications. Network coding has been widely studied in video distribution [19,20], P2P content distribution [21,22], network security [23,24] and other fields. Although the theoretical research on network coding has become mature, few studies are focusing on the application of network coding. The main reason is that the intermediate nodes of traditional networks, such as switch and router, cannot perform re-encoding operations, which has generated significant difficulty in its practical applications.

The network coding technology has outstanding performance on the aspects of improving transmission reliability and increasing the network throughput [25,26,27,28,29]. In a D2D network, the devices have powerful computation ability, so it could be regarded as an intermediate node to perform the re-encoding operation. Therefore, the difficulty of implementing re-encoding operation in traditional networks no longer exists in D2D networks, which implies that the merge of these two technologies has good prospects.

This paper proposes an RLNC-based video transmission scheme for the D2D communication environment. In this scheme, the re-encoding operation is allocated to other nodes during the transmission. The source node sends the encoded video file to the destination nodes through multicast connections. After receiving the data, the destination nodes perform re-encoding operations, and then the re-encoded packets are sent back to the network. Finally, the nearby nodes can obtain and decode the original data through the re-encoded packets. Both the simulation results and actual experimental results showed that the proposed scheme could reduce the computation overhead of the source node, balance the computation load, and reduce the delay caused by the encoding and decoding operation.

The remaining of the paper is organized as follows. In Section 2, we introduce some previous studies related to our research; in Section 3, the system model of the proposed scheme is introduced; in Section 4, the performance of the proposed scheme is analyzed through experiments; finally, in Section 5, we conclude our work.

## 2. Related Work

There are some previous researches which apply network coding in the D2D networks for video transmission [30,31,32]. To improve the quality of service of real-time scalable video without additional infrastructure, Yan et al. [30] proposed to use network coding to assist cooperative transmission. In their scheme, the cooperative transmission is assisted by scheduling coding, which can transmit the scalable real-time video flow to multiple devices. As a result, it could maximally improve video quality. They adopted opportunistic network coding because it does not have to wait for the reception of all linearly independent packets, which can significantly reduce the latency for decoding packets with deadlines. However, opportunistic network coding does not give full play to the computing power of intermediate nodes. Our paper makes full use of the computing power of intermediate nodes and reduces the computing overhead of source nodes by carrying out the re-encoding operation at intermediate nodes. Besides, they studied the QoS by evaluating the decoding quality of video frames. In this paper, both the decoding quality of frames and transmission delay are analyzed.

Karim et al. [31] studied how to use IDNC (Instantly Decodable Network Coding) in the D2D environment to distribute the real-time video sequences to wireless equipment for cooperative transmission. To solve the conflict caused by the synchronous transmission of multiple devices, they built an IDNC-based scheme to represent all feasible coding and non-conflict transmission decisions in a uniform framework. The simulation results of real-time video sequences show that compared with existing IDNC algorithms, their algorithm can improve the quality of the received video. IDNC is essentially a network coding scheme over Galois Field (GF) with size 2, which makes the linear correlation of the received data packets higher, thus increasing the number of useless packets. When RLNC is used, it works over GF(256), the linear correlation of received data packets is far lower than that over GF(2). Moreover, the receivers in their scheme obtain the complete data through the exchange of data between intermediate nodes. In this paper, the source node and destination nodes will automatically calculate the redundancy according to the network conditions and actively send repair packets to obtain the complete data.

Xu et al. [32] proposed a network-coding-based multimedia broadcasting retransmission scheme in D2D networks. Based on the built IDNC diagram, they designed an optimal broadcasting strategy for bulk transmission and sliding window transmission, and proposed a heuristic maximum weight selection algorithm based on network coding (MWSA-NC) to reduce the complexity of optimal search algorithm. The simulation results show that compared to the traditional scheme, their algorithm can improve the system throughput and reduce distortion of video flow. IDNC broadcasting strategy and coding selection algorithm improve some performance for network transmission, but this scheme has coding conflict and transmission conflict between terminal nodes. Although they construct a new IDNC graph to solve the conflict, it inevitably increases some computing burden. In our scheme, we gain performance benefits by re-encoding intermediate nodes, and effectively reduce the computational overhead of source nodes.

Wang et al. [33] combined the RLNC technology with video real-time transmission technology to implement a network-coding-based video conferencing system (NCVCS). The system can not only enable multiple people to have an online video conference but also guarantee video quality. In NCVCS, they introduced a coding server with high performance to perform the re-encoding operation. In this paper, the scheme is designed for mobile devices in D2D communications. Currently, most mobile devices have powerful computational abilities. Therefore, in this multimedia multicast scheme, the re-encoding operation is performed on mobile devices.

The contribution can be summarized as follows. Firstly, we establish a re-encoding model in the multi-hop D2D communication network, which can allocate the computing overhead of the source node to other destination nodes to achieve load balancing. Secondly, we design some algorithms for D2D networks, which can effectively improve the Successful Decoding Rate (SDR). Finally, we built a testbed to evaluate the efficiency of the proposed RLNC-based video transmission scheme. The experimental results show that in addition to guaranteeing the video transmission quality, the proposed scheme also has lower coding latency and balanced computational overhead.

## 3. System Model

### 3.1. Network Model

**Figure 1:**
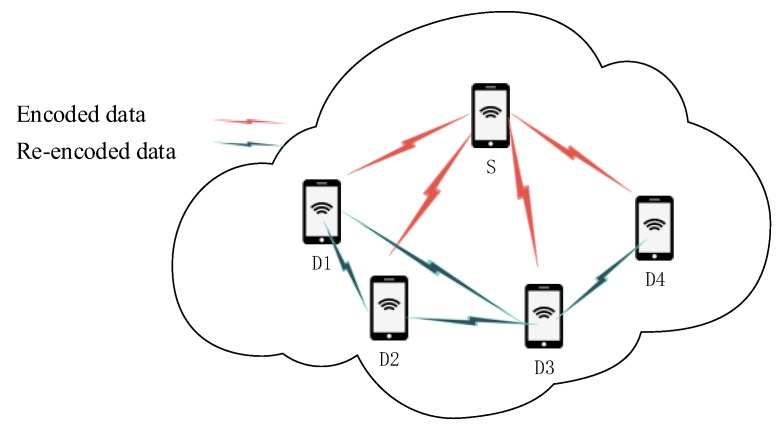
Network model.

In the wireless multi-hop multicast network, as shown in Figure 1, source node *S* encodes the data that needs to be transmitted and then sends it out through a multicast connection. The destination nodes ${(D}_{1}{,\;D}_{2}{,\;D}_{3}{,\;D}_{4})$ perform re-encoding operations after receiving part of the data, and then send the re-encoded packets to the surrounding nodes. To recover the original data, the mobile devices working as destination nodes only need to receive a certain number of linearly independent packets.

Different from the traditional replication-based network transmission method, the network-coding-based transmission method needs to process the data slices before transmission. The source node divides the video frame with length *L* to *K* slices with equal size, each slice has a fixed length of L/K, and when it cannot provide exact division, some zero elements are appended to the end of the last slice.

Each video frame is divided into *K* data slices ${P(p}_{1}{,\;p}_{2}{,\;p}_{3}{,\ldots \;p}_{K})$; then, generate an n–by-K random matrix ${M(a}_{i1}{,\;a}_{i2}{,\;a}_{i3}{,\ldots \;a}_{\mathrm{iK}})(1\leq i\leq n)$ from finite field GF(2^q), at last, multiply the random matrix *M* with data slices to generate *n* encoded slices $Y_{1}{,\;Y}_{2}{,\;Y}_{3}{,\ldots \;Y}_{n}$ with the same size, as shown in Equation (1), where n≥K. When n=K, it implies that there is no redundant data; when n>K, n−K is the number of redundant data slices. Since the scheme in this paper reduces the computational overhead of the source node through the re-encoding operation, we can reduce the redundancy as much as possible according to the network condition. After the encoding operation, all data slices $Y_{i}(i\geq K)$ are linearly independent with a high probability [34].

(1) $$\left[\begin{array}{c} Y_{1}\\ Y_{2}\\ \vdots \\ Y_{n} \end{array}\right]{=\mathrm{MP}}^{T}=\left[\begin{array}{cccc} a_{11} & a_{12} & \cdots & a_{1K}\\ a_{21} & a_{22} & \cdots & a_{2K}\\ \vdots & \vdots & \ddots & \vdots \\ a_{n1} & a_{n2} & \cdots & a_{\mathrm{nK}} \end{array}\right]\left[\begin{array}{c} p_{1}\\ p_{2}\\ \vdots \\ p_{K} \end{array}\right]$$

The source node mixes the encoded data slices with corresponding row vectors in random matrix *M*, and then sends out the newly generated packets. The destination nodes conduct a re-encoding operation after receiving sufficient linearly independent packets. During the re-encoding process, a t-by-h random matrix ${R(b}_{j1}{,\;b}_{j2}{,\;b}_{j3}{,\ldots \;b}_{jh})(1\leq j\leq t)$ is generated. h(h≤K) is the number of received data slices; *t* is the number of data slices generated after re-encoding the *h* data slices; then, multiply the random matrix with the matrix data $P_{M}{(a}_{i+1}{,\;a}_{i+2}{,\;a}_{i+3}{,\ldots \;a}_{i+h})$ in *h* data slices and the encoded data $P_{Y}\left(Y_{i+1}{,\;Y}_{i+2}{,\;Y}_{i+3}{,\ldots \;Y}_{i+h}\right)$ respectively, and *t* re-encoded data slices ${(X}_{t}{,Z}_{t})$ are further generated, in which, $X_{t}\left(c_{i+1}{,\;c}_{i+2}{,\;c}_{i+3}{,\ldots \;c}_{i+h}\right)$ is the corresponding random matrix of re-encoded data, as shown in Equation (2). Finally, the destination nodes send out the data obtained after re-encoding.

(2) $$\left[\begin{array}{c} X_{1\;}Z_{1}\\ X_{2\;}Z_{2}\\ \vdots \;\vdots \\ X_{t\;}Z_{t} \end{array}\right]=R{\left(P_{M}P_{Y}\right)}^{T}=\left[\begin{array}{cccc} b_{11} & b_{12} & \cdots & b_{1h}\\ b_{21} & b_{22} & \cdots & b_{2h}\\ \vdots & \vdots & \ddots & \vdots \\ b_{t1} & b_{t2} & \cdots & b_{\mathrm{th}} \end{array}\right]\left[\begin{array}{c} a_{i+1}{\;Y}_{i+1}\\ a_{i+2}{\;Y}_{i+2}\\ \vdots \;\vdots \\ a_{i+h}{\;Y}_{i+h} \end{array}\right]$$

The destination nodes will conduct decoding operation after receiving *K* linearly independent data slices. These *K* data slices could be the initially encoded data slices, or the combination of the initially encoded slices and re-encoded data slices. During the decoding operation, the destination nodes extract random matrix and encoded data from the packets respectively; then, conduct matrix inversion operation; finally, multiply the obtained inverse matrix with encoded data slices to recover the original data, as shown in Equation (3). Some of the data in the matrix may be re-encoded data ${(X}_{t}{,Z}_{t})$.

(3) $$\left[\begin{array}{c} p_{1}\\ p_{2}\\ \vdots \\ p_{K} \end{array}\right]={\left[\begin{array}{cccc} a_{11} & a_{12} & \cdots & a_{1K}\\ a_{21} & a_{22} & \cdots & a_{2K}\\ \vdots & \vdots & \ddots & \vdots \\ c_{K1} & c_{K2} & \cdots & c_{\mathrm{KK}} \end{array}\right]}^{-1}\left[\begin{array}{c} Y_{1}\\ Y_{2}\\ \vdots \\ Z_{K} \end{array}\right]$$

According to related research [31], when the finite field has a size of GF(256), the balance between memory overhead and computational efficiency can be best maintained, so in this paper, we set the size of the finite field at GF(256).

### 3.2. Analysis of Probability Model

D2D communication is susceptible to the influence of equipments working in the same frequency and base station, which will reduce the reliability of data transmission. Assume the bit error rate of communication between different devices is $P_{b}$, the packet loss rate Q and the length of each transmitted packet is L/K, we can obtain the successful transmission probability of a single packet as p, as shown in Equation (4).

(4) $${p=(1-p}_{b}{)}^{L/K}$$

The probability that each packet can be correctly received by the destination node is d, as shown in Equation (5).

(5) $${d=(1-Q)(1-p}_{b}{)}^{L/K}$$

During the video transmission process, each video frame is divided into *K* packets for transmission. The probability that a group of *K* packets can all reach the destination node and be correctly received is e, as shown in Equation (6).

(6) $${e=[(1-Q)(1-p}_{b}{)}^{L/K}{]}^{K}$$

During transmission under the traditional replication-based mode, the probability of each destination node successfully recovering the original video frame is e. Then, when the redundancy is set at r times, the total number of transmitted packets is (r+1)×K, then, the probability of each destination node successfully recovering the original video frame will become $t_{1}$, as shown in Equation (7).

(7) $$t_{1}=(1-{\left(1-d\right)}^{\left(r+1\right)}{)}^{K}$$

In this process, the probability that one packet is not correctly received for (r+1) times is ${\left(1-d\right)}^{\left(r+1\right)}$; the probability that at least one complete packet is successfully received is $(1-{\left(1-d\right)}^{\left(r+1\right)})$.

Compared to the traditional scheme, in the RLNC-based transmission scheme, each packet after encoding is linearly independent with a high probability, so any packet received by each node is effective [10]. When the total number of encoded packets is *A*, the number of redundant packets is (A−K). Therefore, in the RLNC-based scheme, the probability of each node successfully recovering the original data is $t_{2}$, as shown in Equation (8).

(8) $$t_{2}(K\leq h\leq A)=\underset{h}{\sum }\left(\begin{array}{c} h\\ A \end{array}\right)d^{h}{\left(1-d\right)}^{A-h}$$

h stands for the number of received packets. The node only needs to receive K packets to decode and recover the original video frame.

In the transmission scheme proposed in this paper, the re-encoding operation is conducted at other destination nodes. Assume there are n destination nodes, we can obtain the redundancy of R, as shown in Equation (9).

(9) $${R=n[(1-Q)(1-p}_{b}{)}^{L/K}{]}^{K}$$

Then, the probability for other destination nodes to obtain the original data is $t_{3}$, as shown in Equation (10).

(10) $$t_{3}(K\leq h\leq K+R)=\underset{h}{\sum }\left(\begin{array}{c} h\\ K+R \end{array}\right)d^{h}{\left(1-d\right)}^{K+R-h}$$

### 3.3. Coding Scheme

According to the principle of linear network coding, each destination node must obtain sufficient data slices to conduct a decoding operation. While in an unreliable network transmission environment, packet loss is inevitable. Therefore, we need to design encoding and decoding algorithms to improve this situation.

We designed a coding scheme, which can improve the SDR through the re-encoding of data slices received by other destination nodes. When a destination node receives enough packets, it calculates the number of received packets that belong to the same video frame. The node conducts a re-encoding operation after receiving enough packets that belong to this video frame; then, the re-encoded packets will be sent out; next, the node conducts decoding operation. The surrounding nodes can recover the original data through the combination of encoded and re-encoded packets. Because complete packets are encoded, it can ensure the effectiveness of re-encoded packets. When there are many destination nodes, the decoding efficiency can be significantly improved. During the process in which the destination node is receiving the re-encoded packets from other destination nodes, it could not always be at a waiting state. After all the data belongs to the next frame arrives, the destination node immediately stops the decoding operation of the previous frame.

To deal with different network conditions, we design an encoding algorithm for the source node. In Algorithm 1, During the encoding process, the source node first obtains the value of current signal strength V, and then dynamically set the redundancy according to the V. When the signal strength is greater than −60 dBm, it indicates that the wireless network signal is good, so we use this value as a threshold. When the signal strength is lower than −60 dBm, we will increase the redundancy according to the measured signal strength. This algorithm ensures that the destination node can get enough encoded packets.

After the encoding process is finished, the encoded data will be packaged and sent. We record the number of the frame in the header of each packet to prevent repeated reception and encoding. When we encapsulate a package, we pack the encoded data and the corresponding row vectors into the package. When a node receives a data packet, it will check whether the data packet is linearly independent of the packets that have been received. If it is, it discards the data packet; otherwise, it accepts the packet. In this way, nodes can avoid receiving duplicate or linearly dependent data packets.

**Algorithm 1** Encoding strategy at the source node1: Destination nodes obtain the value of current signal strength V. 2: Destination nodes send packets ***pkg1*** to the source node S. 3: S receives some packets ***pkg1*** from the destination nodes.4: S obtains the value of V from ***pkg1***. 5: S works out the mean value ***Avg*** of ***V***. 6: **If**
Avg≥−60
7:  Set the redundancy value r to ***0***. 8: **Else** 9:  Set the redundancy value r to ***(−60−Avg)/20***.10: **Endif**11: S divides a data frame F into K
**slices.**12: S generates a (K+r)–by-K random matrix M. 13: S generates encoded data Y with M and F. 14: The encoded packets consisting of row vectors of M and Y will be generated. 15: S sends encoded packets to the network.

To improve decoding efficiency, we designed a re-encoding algorithm and a decoding algorithm. In the algorithms, N stands for the frame number, which is placed in the header of the data packet to distinguish different frames. When nodes receive data packets, they need to determine whether the frame number N in the data packet is the same as the frame number being received. Packets with the same frame number will be accepted; otherwise, the package will be dropped. Flag is the flag of the current frame, RFlag is the flag of re-encoding, and DFlag is the flag of decoding. These flags are recorded in each destination node and are used to mark whether frame data is available. Besides, $P_{i}$ is a row vector of the random matrix, and $Y_{i}$ is an encoded data slice corresponding to the row vector. G is a random matrix composed of the row vectors in the received data packet, and H is the sum of the encoded data slices that have been received.

**Algorithm 2** Re-encoding strategy1: Intermediate node T receives a packet pkg2 from S.2: T extracts frame number N from pkg2. 3: **If** N==RFlag 4:  T discards pkg2. 5: **Else**
6:  T extracts row vector $P_{i}$ and encoded data slice $Y_{i}$ from pkg2. 7:  **If**
N==Flag
8:    r1=rank(G) 9:    ${r2=\mathrm{rank}(P}_{i}\cup G)$ 10:    **If**
r2>r1
**do** 11:      Add $P_{i}$ into matrix G.12:      Add $P_{i}$ into existing encoded data H.13:      Count++;14:      **If**
Count==K
15:        Re-encode G, H and compose new data packets pkg. 16:        T sends pkg
**back to the network.** 17:        RFlag=N
18:      **Endif**
19:    **Endif** 20:  **Else**21:    Flag=N
22:     Get K from pkg2 and save the data in it23:    Clear matrix G and encoded data H
24:    Count=0

25:  **Endif**
26: **Endif**

In Algorithm 2, after receiving a packet, the intermediate node first obtains the video frame number N of the packet and then checks whether the frame has been re-encoded. In other words, the intermediate node checks whether the frame number N of the frame already exists in the re-encoding flag RFlag. If number N exists, it means that the node has re-encoded the frame; otherwise, it has not been re-encoded. If the frame has been re-encoded, it will be dropped. Otherwise, it will be recorded. Then we need to determine whether the packet belongs to the current frame. If it does not belong to the current frame, we store the data and mark the frame number. If it does, we verify the availability of the data. If the data is not available, the data packet will be dropped. Otherwise, the data will be saved. Then we check if the amount of data is sufficient. If K linearly independent packets are received, these packets will be re-encoded and sent out.

**Algorithm 3** Decoding strategy at the destination node1: Destination node D receives a packet pkg3.2: D gets frame number N from pkg3. 3: **If**
N==DFlag 4:    D discards pkg3. 5:  **Else** 6:    D gets row vector $P_{i}$ and coded data slice $Y_{i}$ from pkg3. 7:    **If**
N==Flag
8:      r1=rank(G) 9:      ${r2=\mathrm{rank}(P}_{i}\cup G)$ 10:      **If**
r2>r1
**do** 11:        Put $P_{i}$ into G12:        Put $Y_{i}$ into existing encoded data H.13:        Count++;14:        **If**
Count==K
15:          D calculates the inverse matrix $G^{-1}$ of G. 16:          D uses $G^{-1}$ to decode H into raw data P.17:          D sends P to the play module. 18:          DFlag=N 19:        **Endif** 20:      **Endif**21:    **Else**22:      Flag=N
23:        D gets K from pkg3. 24:        Clear matrix ***G*** and encoded data ***H***25:        Count=0
26:        goto step 627: **Endif**

In Algorithm 3, after receiving a packet, the destination node first obtains its video frame number N. The destination node then checks whether N already exists in the decoding flag DFlag. If  exists, it indicates that the frame has been decoded; otherwise, it means that the frame has not been decoded. If the frame is not decoded, the algorithm checks the availability of the data. If the data is useful, it will be stored in the memory for further decoding.

After loading the encoded data slice, the integrity of the data should be checked first. If the data slice is enough, the random matrix in it will be extracted, and then the inverse matrix of the random matrix should be calculated. After that, the inverse matrix and the encoded data should be multiplied to recover the original data, namely the original video frame in the H.264 format. Finally, the data frame in the H.264 format is sent to the play module. If there is insufficient data or the decoding operation has been completed, the next packet is immediately fetched.

## 4. System Evaluation

To evaluate the performance of the proposed scheme, we experimented in a simulated scenario and a real scenario, respectively. First of all, we evaluate the feasibility of the scheme on the two aspects of network coding efficiency and delay distribution. Further, we analyze the performance with the SDR and Peak Signal to Noise Ratio (PSNR) and compare the performance with the traditional replication-based scheme and the RLNC-based scheme.

**Figure 2:**
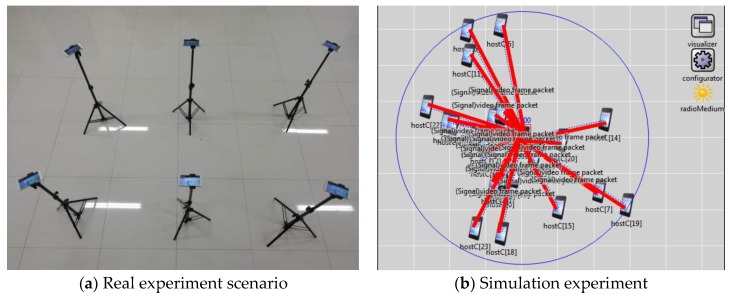
Experiment scenarios.

The experimental parameter settings are listed in Table 1:

Because the D2D communication module hasn’t been implemented in commercial mobile devices at the current stage, we used the WiFi Direct technology to implement device-to-device communication between different devices. In the experiment, the video transmission is conducted in the form of a UDP multicast by adopting the IEEE 802.11g protocol. The experimental scenarios for this scheme are shown in Figure 2.

### 4.1. Network Coding Efficiency

When network coding is conducted during the transmission process, it generates some computation overhead. Because mobile devices have lower computing power than computers and servers, it is particularly important to evaluate the network coding efficiency of mobile devices. If the coding delay is too long, the performance gain will decline, which will result in poor user experience.

**Figure 3:**
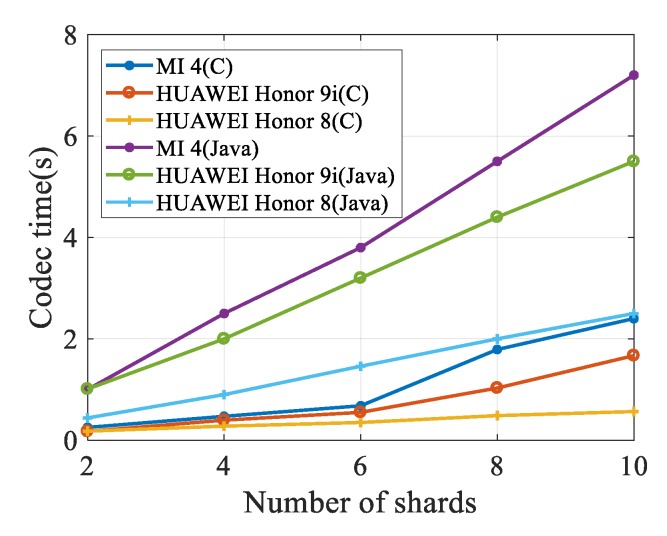
Encoding Efficiency in Different Mobile Devices.

In this section, we evaluate the data coding capacity of different mobile devices. In the experiment, the size of the testing file is 10 MB, and generation size *K* is in the value range from 2 to 10. Most previous tests in the Android operating system were conducted by using the Java language, the encoding and decoding took more than 2 seconds on average, and this is unacceptable to the users. To reduce delay, we designed and implemented a network coding module with Java native interface, which is implemented with native C language. In accordance with Figure 3, we observe that our scheme requires much less time for encoding and decoding than merely using the Java program. With the increase of *K*, the device needs more time for network coding, which is mainly due to the increased coding complexity caused by a higher coding dimension.

With the introduction of the JNI library, the consumption of encoding time is significantly reduced, which is beneficial for the destination nodes to carry out re-encoding operation after receiving data. The experimental results show that it is feasible to research applications based on network coding at current commercial mobile devices.

Because the main computational work of the scheme proposed in this paper is matrix multiplication, the time complexity of our scheme is O (*n*^3^), and the space complexity is O (*n*^2^).

### 4.2. Analysis of SDR

**Figure 4:**
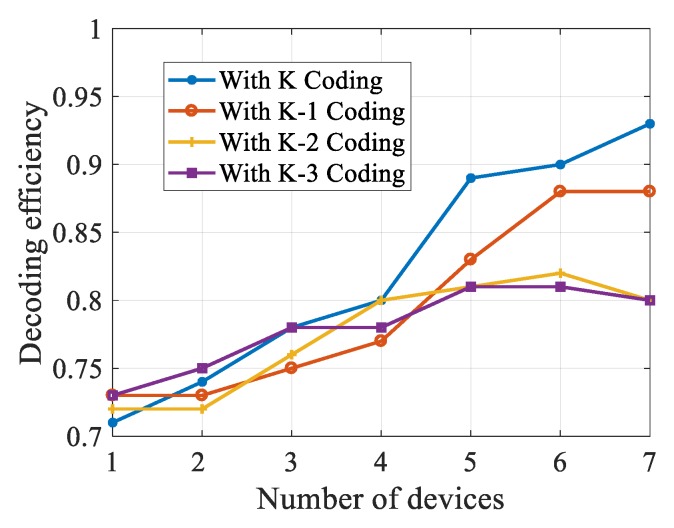
Influence of different re-encoding scheme on successful decoding rate (SDR).

In the experiment, first, we tested how many packets do the destination nodes need to receive before re-encoding to achieve higher efficiency. When performing a re-encoding operation, the nodes need to determine when to re-encode the received packets. The value can be at most *K*, at least 2. When the number of fragments is large, the linearly mixed data has high availability; when the number of packets is too small, the availability of the combined data is low, and the decoding delay will increase significantly. The experimental results are shown in Figure 4.

In Figure 4, we observe that the best performance can be achieved when the nodes conduct the re-encoding operation after receiving the complete data because the packets obtained after re-encoding will be more useful. If the nodes conduct re-encoding operation after receiving partial data when other nodes receive the re-encoded packet, they need to check whether the re-encoded packet is linearly independent to their packets, which will increase the decoding delay. Furthermore, if the re-encoding operation is conducted after receiving partial data, it will increase the nodes of re-encoded data slices, which will cause channel jam and significantly reduce the transmission efficiency. According to the diagram, it can be seen that if the re-encoding operation is conducted after receiving complete data, the SDR will decrease. Therefore, we choose to conduct a re-encoding operation after the destination node has received *K* packets.

**Figure 5:**
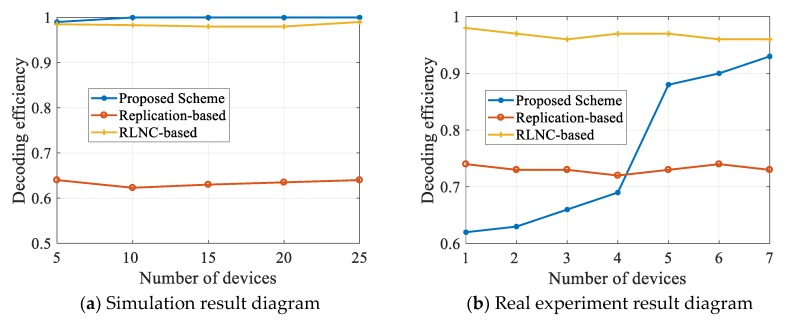
Influence of equipment quantity on SDR.

The relationship between video SDR and the number of destination nodes is shown in Figure 5. Simulation experiments are performed at a 5% loss rate, while real experiments are performed under normal circumstances. In these two experiments, the redundancy value of the proposed scheme is 0, and the redundancy values of the other two schemes are 3. According to the simulation experiments, when the number of mobile devices is large, the SDR of the proposed scheme is almost 1, while the SDR of the RLNC-based scheme is about 0.98, and the replication-based scheme is only about 0.64. Besides, our solution was implemented without redundancy.

In real experiments, when there are fewer destination nodes, SDR is lower than 0.7. However, when the mobile phone number is 5, the SDR reaches 0.9. With the increase of mobile phone numbers, the SDR could reach around 0.93. It can be known from the simulation results that when the number of devices is further increased, the decoding efficiency of the proposed scheme will be higher. In the other two schemes, the mobile phone number does not have any influence on the SDR, and the SDR is maintained the same. Also, in the traditional replication-based scheme, high redundancy is required in advance. Otherwise, the SDR will become very low, which in turn incurs the poor decoding quality.

**Figure 6:**
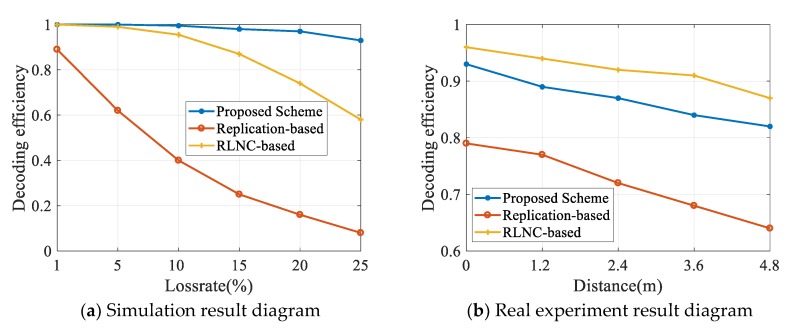
Influence of loss rate and distance on SDR.

In the real scenario, the destination nodes are not always next to the source node, and different distances will lead to different packet loss rates. Therefore, we must consider the effect of distance on wireless data transmission. To this end, we have also conducted corresponding experiments. We performed simulation experiments on the performance of the three schemes under different packet loss rates and conducted real experiments on the effect of distance on the SDR. The results are shown in Figure 6. In the simulation experiment, the number of mobile devices is 25, and the redundancy value of RLNC scheme and the replication-based scheme is set at 3. From the simulation experiment diagram, we observe that the SDR of the three schemes decreases to varying degrees with the increase of the packet loss rate. The scheme proposed in this paper has a tiny decline, the RLNC-based scheme has a moderate decline, and the replication-based scheme has a significant decline. The solution in this paper can still maintain a very high SDR under high packet loss. Therefore, it can be proved that the proposed scheme has the most robust anti-interference ability.

In the real experiment, the analysis result of the relationship between video SDR and distance is as shown in Figure 6b. With the increase of transmission distance, the packet loss rate increases. In Figure 6b, the SDR of the replication-based scheme declines significantly with the increase of distance. In contrast, the SDR of the RLNC-based scheme and our scheme has a gentle decline, which indicates that distance only has a small influence on these two network coding schemes. The reason for this result is that the coding operation has increased the independence among packets and improved the anti-interference ability.

**Figure 7:**
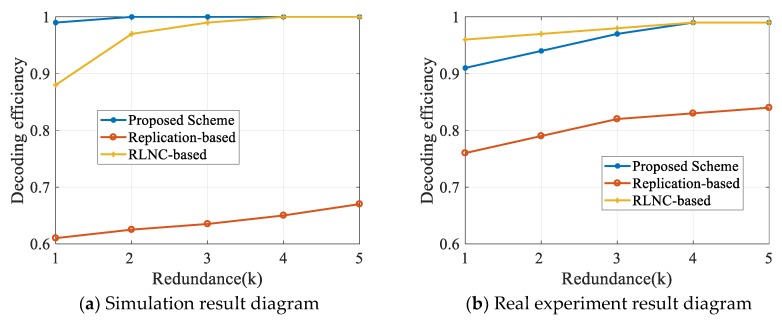
Influence of redundancy on SDR.

The three schemes in our experiments all use redundant operations to improve transmission reliability, so we tested the impact of the amount of redundant data on SDR. The experimental results are shown in Figure 7. In the simulation experiments, we set the packet loss rate at 5% and the number of mobile devices at 25. From the results, we observe that the scheme proposed in this paper and the RLNC-based scheme can easily reach 1 with a small amount of redundancy. However, the SDR of the replication-based scheme only increased slightly.

It has a similar result in real experiments. The two schemes based on linear network coding can both achieve an SDR above 0.9, and with the increase of redundancy, the SDR could get close to 1. Although the SDR of the replication-based scheme increases with the increase of redundancy, the improvement rate is limited, mainly because the replication-based scheme tends to receive more repeated and invalid packets.

### 4.3. PSNR Video Quality Analysis

**Figure 8:**
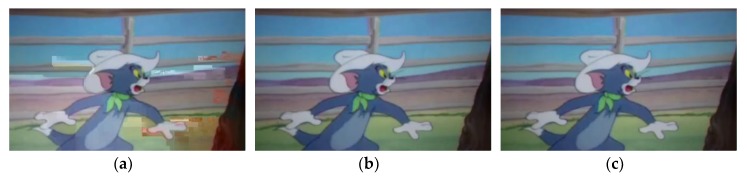
Experimental result diagram: **(a)** Description of Replication-based scheme; **(b)** Description of Proposed scheme; **(c)** Description of Random Linear Network Coding (RLNC)-based scheme.

The actual performance of the three schemes in this paper is shown in Figure 8. The PSNR of the replication-based scheme is 24.6, and the PSNR of the other two schemes is 100. The image quality transmitted by the two schemes based on network coding is higher than that based on the replication. The transmission quality of the proposed scheme is similar to the traditional RLNC-based scheme.

The overall result of video transmission is shown in Table 2. In the table there are the number of sent frames and the number of decoded frames and the quality of the transmitted picture.

**Figure 9:**
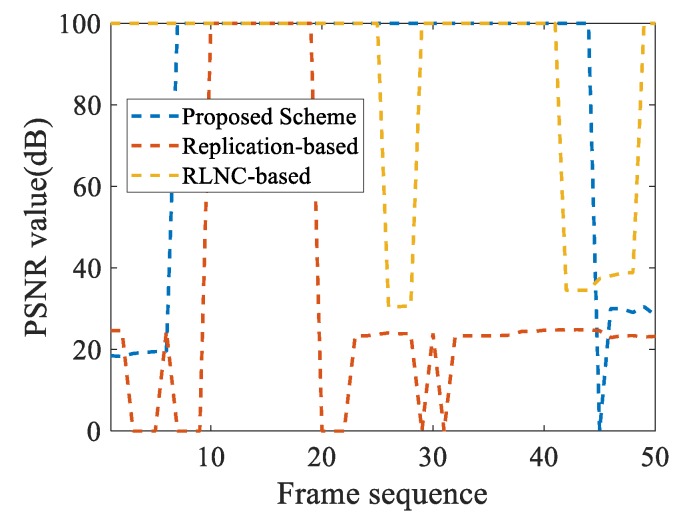
Analysis of video quality.

The quality of the video is an essential criterion for testing the reliability of transmission, so it needs to be focused on experiments. In the experiment, we analyze the video quality of the three schemes with PSNR as the criterion, as shown in Figure 9. PSNR is a primary video quality evaluation indicators, and its value ranges from 0 to infinity. The PSNR value indicates the degree of distortion of the picture. The larger the value is, the smaller the distortion is. Generally, when the PSNR value is higher than 50, it means that the picture quality is excellent. We consider that the picture is almost completely restored when the PSNR value exceeds 100. So we set all values higher than 100 to 100. Moreover, when the data frame is lost, the PSNR value is set to 0. Therefore, in the figure, the value of PSNR ranges from 0 to 100.

According to the diagram, the replication-based scheme has the most inadequate video decoding quality and the least successfully decoded frames, and the other two schemes have similar decoding and recovery performance. Experimental results show that the average PSNR value of the replication-based recovery rate is only 45. However, the recovery rate of the proposed scheme and the RLNC-based scheme are 85 and 84. Compared with the replication-based scheme, the proposed scheme can significantly improve the frame quality. Moreover, the PSNR value of the decoded frames is slightly better than that of the traditional RLNC scheme, but the number of decoded frames in the proposed scheme is less than that of the RLNC scheme. Therefore, we consider that the video quality of the proposed scheme is comparable to that of the traditional RLNC scheme.

### 4.4. Transmission Delay Analysis

During the experiment, operation at different stages will cause some delay, such as the encoding delays, transmission delay, decoding delay, etc., so we analyzed the delay caused in these stages. We recorded the time before and after different operations of the video frame, including operations such as reading, transmitting, encoding, and decoding. These timing points are then used to calculate the duration of different operations accurately. We summed up the time needed for various operations of all frames. Finally, we calculated the average time required for each frame after multiple measurements. The delay distribution is shown in Table 3:

In Table 3, all delays are average delays. The delays caused by the reading operation are consistent; the replication-based transmission method does not have any encoding delay; the scheme proposed in this paper requires two milliseconds less encoding time than the RLNC-based scheme. During the transmission process, the RLNC-based scheme has the most prolonged delay. During the decoding process, the replication-based method takes the least time, which only requires 9 ms; our proposed scheme requires 11.5 ms; the RLNC-based scheme takes 11.1 ms. In general, the replication-based transmission method has the shortest delay, while the RLNC-based transmission method involves the most prolonged delay. Compared with the traditional RLNC-based scheme, our proposed scheme can reduce codec delay by about 8%.

### 4.5. Computational Load Analysis

**Figure 10:**
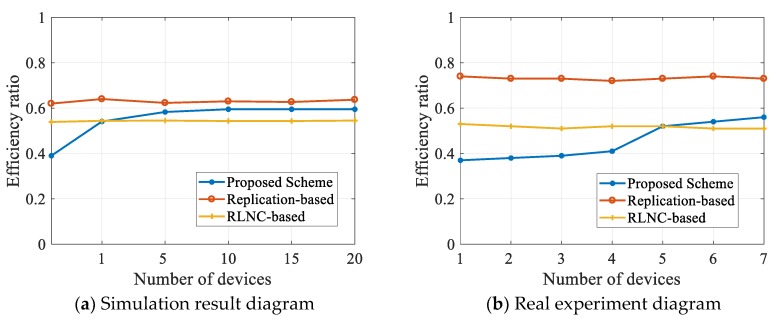
Analysis of utilization.

Finally, we also evaluated the computation efficiency of each scheme. We recorded the total time and useful time of sending all data, then calculated the SDR after transmission. The useful time is divided by the total time and then multiplied by SDR. Further, we could obtain the efficiency ratios. We took the average after several measurements, and finally obtained the results, which are shown in Figure 10.

In accordance with Figure 10, no matter in the simulation experiment or real experiment, the replication-based scheme has the highest utilization. This is because the scheme does not perform redundant operations, but the video transmission quality is deficient in this case. The proposed scheme in the initial phase is slightly lower than the traditional scheme based on RLNC. With the increase in the number of mobile phones, the utilization rate of our scheme slowly improves and soon outperforms the traditional RLNC scheme.

**Figure 11:**
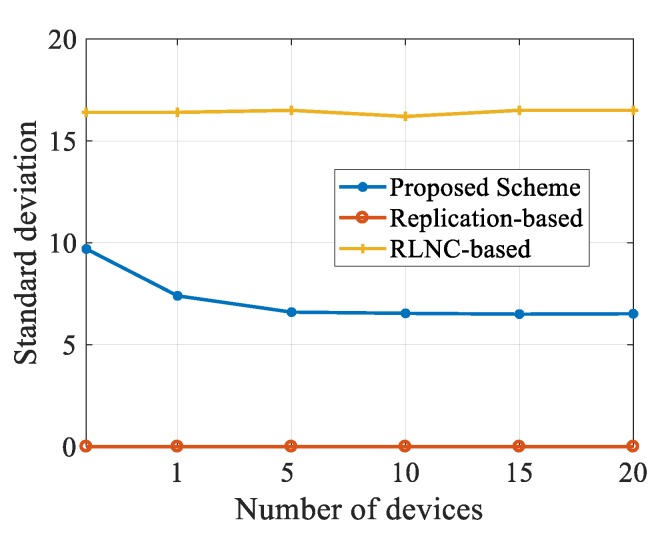
Analysis of load balance.

To evaluate whether the schemes in this paper can achieve the purpose of load balancing, we calculated the average load value of each scheme. In the experiment, the redundancy at the source nodes in other schemes is set at 3. In our scheme, the value of redundancy is set at 0. The experimental results are shown in Figure 11.

In the experiment, we calculate the increased load of encoding and decoding. Because the replication-based scheme has no codec operation, the load value of this scheme is always zero. From Figure 11, the average load value of the nodes in our scheme is only half that of the RLNC-based scheme, and this is because the redundant operations of the source nodes are reduced. Besides, as the number of nodes increases, the standard deviation of our scheme declines.

## 5. Conclusions

To balance the computation load of the nodes in the networks, reduce the transmission delay, and improve transmission reliability, we propose a new RLNC-based video transmission optimization scheme for multi-hop D2D networks. In this scheme, some encoding operations are allocated to various destination nodes, which can not only alleviate the computation burden at the source node but also guarantee the video quality. However, the re-encoding and multicasting operation considered in our scheme requires further optimization. we will study how to reduce the delay further and improve the successful video delivery rate in future work.

## Figures and Tables

**Table 1:** Experiment parameters.

Item	Value
Mobile device	MI4LTE
Android version	6.0
Memory size	2 GB
Video size	6 MB
Package size	1500 Bytes
Number of frames	960
Number of packets	8502
Video length	32s
Video Format	H.264
Transmission method	Multicast
Transmission distance	0–5 m
Transmission protocol	IEEE 802.11g
Number of devices	7

**Table 2:** Transmission situation.

Parameter	Replication-Based	RLNC-Based	Our Scheme
Total	960	960	960
Decoding quantity	730	926	897
Average PSNR value	45	84	85

**Table 3:** Delay distribution.

Operation	Replication-Based	RLNC-Based	Proposed Scheme
Reading	10 ms	10 ms	10 ms
Encoding	0 ms	10.2 ms	8.2 ms
Transmitting	2.2 ms	2.5 ms	2.2 ms
Decoding	9 ms	11.1 ms	11.5 ms
